# Transforming cancer care in Australia: The dawn of particle therapy

**DOI:** 10.1002/jmrs.784

**Published:** 2024-04-01

**Authors:** Peter Gorayski, Frank Saran

**Affiliations:** ^1^ Royal Adelaide Hospital Adelaide South Australia Australia; ^2^ South Australia Health and Medical Research Institute Adelaide South Australia Australia; ^3^ Australian Bragg Centre for Proton Therapy and Research Adelaide South Australia Australia; ^4^ Allied Health and Human Performance Academic Unit University of South Australia Adelaide South Australia Australia

## Abstract

The nearing completion of the Australian Bragg Centre for Proton Therapy and Research marks a transformative leap in cancer care in Australia. Highlighting the precision and potential of particle therapy in reducing long‐term side effects, particularly in paediatric and rare cancers, this editorial underscores Australia's commitment to integrating this innovative modality into national healthcare, despite challenges in accessibility and cost.

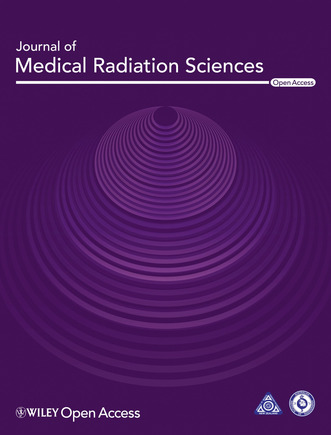

The development of the Australian Bragg Centre for Proton Therapy and Research (ABCPTR) heralds a significant shift in Australian healthcare, marking the introduction of particle therapy as a cutting‐edge treatment option for cancer patients. This transformation is celebrated in this special edition of the Journal of Medical Radiation Sciences, which showcases the Australian radiation oncology community's united effort to adopt this ground‐breaking treatment modality.

Particle therapy is a form of external beam radiation therapy that uses ionising particles, such as protons, neutrons or carbon ions, to target and destroy cancer cells with precision while minimising damage to surrounding healthy tissue. Proton therapy, leveraging the unique properties of protons, contrasts sharply with traditional photon‐based (x‐ray) radiation therapy. The ‘Bragg peak’ phenomenon allows protons to deposit their maximum energy directly into the tumour with minimal exit dose, sparing surrounding healthy tissue. This precision is particularly beneficial in paediatric cases and adults with rare cancers, where reducing long‐term side effects is crucial. The first patient treatment with proton therapy in 1954 at Berkeley, California, set the stage for decades of advancements, culminating in a substantial body of clinical evidence, underpinning its increasing utility in cancer care.^1^ Studies have demonstrated particle therapy's role in reducing secondary neoplasms and improving cognitive outcomes in paediatric patients, among others, propelling the global expansion of particle therapy facilities, as noted by the Particle Therapy Co‐Operative Group (PTCOG).^2^, ^3^

Australia acknowledges its tardiness in proton therapy, yet the recognition of its importance is evident through national working groups such as the Royal Australian and New Zealand College of Radiologists (RANZCR), the Trans‐Tasman Radiation Oncology Group (TROG), the Australian Society of Medical Imaging and Radiation Therapy (ASMIRT), and the Australasian College of Physical Scientists and Engineers in Medicine (ACPSEM). Australian expertise in particle therapy is undeniable, as reflected in the breadth of research articles in this special edition. These contributions cover a wide range of topics, signifying a deep understanding and proficiency in particle therapy across the country.

However, the pathway to integrating particle therapy into national healthcare is fraught with challenges. The Medical Services Advisory Committee (MSAC) has currently approved a very limited set of reimbursable indications for proton therapy, constraining its application compared to countries like the United States, the United Kingdom, Germany and Japan.^4^ With over 150,000 Australians diagnosed with cancer annually and about 38% receiving radiation therapy (despite an optimal utilisation target of 52%), just over 440 patients per year are predicted to qualify for proton therapy under the current reimbursement model.^5^, ^6^ This figure includes a significant portion of paediatric, adolescent and young adult (AYA) cases, leaving only a small percentage of adult patients eligible for this treatment.

The potential for expanding proton therapy indications exists, mirroring international standards and evidence for conditions such as head and neck cancers and re‐irradiation scenarios. Yet, the likelihood of a significant broadening of indications in the near term appears slim. This reality underscores a strong desire for particle therapy facilities across Australia's major states, driven by the national healthcare objective of delivering cancer care closer to patients' homes. This is especially critical for First Nations and Torres Strait Islander communities, who face considerable disadvantages due to the vast distances to healthcare facilities.

The establishment and operational costs of a particle therapy facility are high, reflecting the advanced training required for clinicians, medical physicists and radiation therapists and the sophisticated technology involved. These financial considerations, coupled with the limited range of current clinical indications, pose significant challenges. Cancer Australia's national assessment suggests that the justification for a particle therapy facility in every major state is untenable, given Australia's population size and the expected range of treatment indications.^7^

Yet, the role of radiation therapy in cancer treatment, known for its cost‐effectiveness and efficacy compared to say, medical oncology treatments, provides a broader context for evaluating the investment in proton therapy. For example, the cost of a single immunotherapy drug in the US can triple the entire budget for radiation therapy, underscoring the economic viability of investing in particle therapy.

The forthcoming ABCPTR, poised to be Australia's pioneer proton therapy facility, represents more than a national resource; it embodies the potential for enhanced patient outcomes and is accessible to clinicians nationwide through platforms like the Australian Particle Therapy Clinical Quality Registry (ASPIRE) registry. The possibility of expanding access to proton therapy—and potentially to heavy particle therapy—promises to elevate patient care, collaboration and research to unprecedented levels in Australian radiation oncology.

In this special edition of the Journal of Medical Radiation Sciences, the Australian radiation oncology community collectively showcases its significant contributions to the field of particle therapy. Through a diverse range of articles, researchers illustrate the transformative potential of particle therapy, especially for paediatric and rare cancers, by emphasising its precision, efficacy and patient‐centred care principles. Australian publications have previously highlighted proton therapy's capacity to minimise radiation exposure and long‐term side effects in paediatric patients, addressing the ethical considerations of treatment access and eligibility.^8^ Building on this, the creation of the Bragg Consumer Advisory Group (BCAG), as discussed by Penfold M, et al., demonstrates a progressive step towards incorporating patient perspectives into healthcare decision‐making, reinforcing the importance of patient engagement.^9^ Skelton et al. delve into the challenges encountered by families travelling overseas for proton therapy, underscoring the necessity for enhanced support and care coordination.^10^ Mathew et al. explore the advancements in treating clival chordomas with particle therapy, signalling a shift towards more targeted and effective treatment options within Australia's healthcare infrastructure for this rare cancer.^11^ Additionally, initiatives like the Victorian Comprehensive Cancer Centre's comparative planning service for photon and proton therapy and the evaluation of fixed‐beam and gantry‐based proton therapy planning methods used in the treatment of base of skull chordoma highlight the ongoing efforts to enhance local clinical and technical expertise and treatment accessibility.^12^, ^13^ Wood et al.'s scoping review on the impact of the COVID‐19 pandemic on proton therapy services calls for further research to refine patient selection and care strategies.^14^ Furthermore, Thwaites et al.'s discussion on the integration of carbon ion radiation therapy alongside proton therapy facilities points to the clinical and scientific potential to advance cancer treatment research.^15^

As we stand on the brink of integrating particle therapy into Australia's healthcare framework, we celebrate the innovation, collaboration and commitment to patient care showcased in this special edition. The introduction of particle therapy in Australia is not merely a technological milestone but a leap towards evidence based, advanced cancer care utilising particle therapy, heralding a new era in oncology treatment that holds promise for improving oncological outcomes and long term quality of life for many Australian patients.

## CONFLICT OF INTEREST STATEMENT

Both authors declare no conflict of interest.

## Data Availability

Data sharing is not applicable to this article as no new data were created or analyzed in this study.
